# How to discuss about do-not-resuscitate in the intensive care unit?

**DOI:** 10.5935/0103-507X.20190051

**Published:** 2019

**Authors:** Cassiano Teixeira, Paulo Ricardo Cerveira Cardoso

**Affiliations:** 1 Departamento de Medicina Interna e Programa de Pós-Graduação em Ciências da Reabilitação, Universidade Federal de Ciências da Saúde de Porto Alegre - Porto Alegre (RS), Brasil.; 2 Departamento de Medicina Interna, Hospital Moinhos de Vento - Porto Alegre (RS), Brasil.; 3 Unidade de Terapia Intensiva, Hospital São Lucas, Pontifícia Universidade Católica do Rio Grande do Sul - Porto Alegre (RS), Brasil.; 4 Unidade de Terapia Intensiva, Hospital de Clínicas de Porto Alegre, Universidade Federal do Rio Grande do Sul - Porto Alegre (RS), Brasil.

**Keywords:** Ethics, Cardiopulmonary resuscitation, Personal autonomy, Prognosis, Intensive care units

## Abstract

The improvement in cardiopulmonary resuscitation quality has reduced the mortality of individuals treated for cardiac arrest. However, survivors have a high risk of severe brain damage in cases of return of spontaneous circulation. Data suggest that cases of cardiac arrest in critically ill patients with non-shockable rhythms have only a 6% chance of returning of spontaneous circulation, and of these, only one-third recover their autonomy. Should we, therefore, opt for a procedure in which the chance of survival is minimal and the risk of hospital death or severe and definitive brain damage is approximately 70%? Is it worth discussing patient resuscitation in cases of cardiac arrest? Would this discussion bring any benefit to the patients and their family members? Advanced discussions on do-not-resuscitate are based on the ethical principle of respect for patient autonomy, as the wishes of family members and physicians often do not match those of patients. In addition to the issue of autonomy, advanced discussions can help the medical and care team anticipate future problems and, thus, better plan patient care. Our opinion is that discussions regarding the resuscitation of critically ill patients should be performed for all patients within the first 24 to 48 hours after admission to the intensive care unit.

## INTRODUCTION

In recent years, cardiopulmonary resuscitation (CPR) has shown great advances, but both in-hospital^(1-3)^ and out-of-hospital^(4,5)^ cardiac arrest (CA) are associated a reduced probability of survival.^(1,2,4,6)^ In addition, survivors of in-hospital CA who are discharged from the hospital have an 18.5% - 19.2% risk of severe brain damage.^(1)^ In intensive care units (ICUs), approximately 17% of patients who undergo cardiopulmonary resuscitation (CPR) maneuvers show return of spontaneous circulation (ROSC), of which 11.3% (non-shockable CA) and 37.2% (shockable CA) are discharged from the hospital.^(7)^ Notably, approximately 75% of those who are discharged recover their ability to perform activities of daily living (ADLs).^(7)^ However, in this in-hospital scenario, non-shockable CA predominates (75.8%), and the survival rate depends greatly on the hemodynamic condition and degree of organ dysfunction of the patient.^(7)^ It is estimated that patients who use vasopressors who suffer CA on asystole have a 5.9% chance of ROSC and that, of these, only 29.1% recover their ability to perform ADLs.^(7)^

Parallel to this, there is growing literature on the decisions shared by physicians, patients and their relatives,^(8)^ standardization of decisions,^(9)^ and reduction in excess diagnostic and therapeutic interventions.^(10)^ Should we submit a patient to a procedure that offers an immediate 6% chance of survival and an approximate 70% risk of hospital death or severe and definitive brain damage? Based on these data, we should discuss or decide whether patients should be resuscitated when in CA in the ICU. Could this discussion bring any benefit to the patients or their relatives?

## NEUROLOGICAL DAMAGE

Most patients who suffer in-hospital CA die during the initial event, and some die in the first 24 hours after ROSC due to hemodynamic failure.^(11,12)^ However, one of the major problems in this scenario is the high rate of neurological sequelae in the survivors.^(4,13,14)^ The majority of deaths after ROSC are due to hypoxic-ischemic brain injury (46% - 65.2%),^(11,12)^ which results in active removal from life support based on a poor neurological prognosis. Furthermore, there is a 12.6% prevalence of brain death diagnosed, on average, on the third day after ROSC, according to a recent meta-analysis by Sandroni et al.^(14)^ Of patients with ROSC after in-hospital CA, 56.4% - 68% die during hospitalization.^(1)^ We estimate that in more than 50% of cases, the patients progress to brain death or have limited therapeutic support due to the unfavorable neurological prognosis. However, survivors who are discharged from the hospital have a 9.8% - 12.2% risk of severe brain damage [(Cerebral Performance Category - CPC score) 3 (severe cerebral disability), 4 (comatose or in persistent vegetative state) or 5 (brain death)] and a 27.3 to 31% risk of moderate brain damage [CPC = 2 (moderate cerebral performance)].^(1)^ Improvement in the quality of CPR has increased the ROSC rate but does not seem to have affected the rates of severe brain damage.^(1)^

Elderly patients who suffer in-hospital CA are less likely to survive (< 80 years: 27.9%; 80 - 90 years: 20.1%; > 90 years: 15.1%; p < 0.001), especially when they have a higher number of comorbidities.^(15,16)^ Patients who are discharged do not show worse cerebral performance (CPC 1 or 2) when compared to the cerebral performance of younger patients (< 80 years: 92.4%; 80 - 90 years: 92.9%; > 90 years: 87.5%; p = ns).^(15)^

## RESUSCITATION PROGNOSIS

Cardiac arrest that occurs in the hospital is not associated with a higher probability of survival (15.9 - 17%) than that for CA that occurs outside of a hospital environment.^(1,7)^ This is due to the greater presence of comorbidities and the higher probability of non-shockable CA in individuals in a hospital setting.^(1)^ In addition, or in association with this, CA treated in a hospital (except in emergency rooms, cardiac hemodynamics/cardiac electrophysiology rooms and operating rooms) occurs predominantly (in approximately 80% of cases) with non-shockable rhythms,^(1,3,7)^ which have a lower rate of ROSC than does shockable rhythms.^(4)^

In the ICU, the scenario is even less encouraging because patients with acute organ dysfunction are already partially resuscitated using life-support technologies (for example, vasoactive drugs, mechanical ventilation, renal replacement therapy and transfusion of blood products).

A data registry of 411 American hospitals was used to evaluate 51,919 patients who suffered CA while in intensive care; the results indicated that approximately 1 in 6 patients undergoing CPR maneuvers had ROSC.^(7)^ A total of 75.9% of the patients had CA with non-shockable rhythms, only 11.3% of were discharged from the hospital, and 70% recovered their ability to perform ADLs. However, for patients with CA with a shockable rhythms, 37.2% were discharged from the hospital, and 79.8% recovered their ability to perform ADLs.

In patients with hemodynamic impairment who received vasoactive drugs at the time of CA, the rate of ROSC were 22.6% for CA with shockable rhythms and 5.9% for CA with non-shockable rhythms. In addition, 11.4% of patients with shockable rhythms were discharged from the hospital, whereas only 2.1% of patients with non-shockable rhythms were discharged from the hospital.^(7)^ In the present study, a favorable neurological prognosis was observed in approximately 83% of the patients discharged, regardless of the CA rhythm and the presence or absence of shock immediately before CA. The scenario seems to be slightly worse when CA occurs on weekends or during night shifts.^(2)^

In addition to the presence of shock, the need for mechanical ventilation (odds ratio - OR: 0.60; 95% confidence interval - 95%CI 0.56 - 0.63), age ≥ 65 years (OR: 0.77; 95%CI 0.73 - 0.82) and occurrence of CA at night or on the weekend (OR: 0.77; 95%CI 0.72 - 0.81) were independent predictors of reduced patient survival.^(7)^ Notably, patients with metastatic cancer who suffered CA in the ICU had a 1.1% chance of survival.^(7)^ Al-Alwan et al.^(17)^ analyzed MedCare data from 471,962 patients who suffered in-hospital CA; the results revealed that those under mechanical ventilation had lower in-hospital survival [10.1% (95%CI: 9.8% - 10.4%) *versus* 19.2% (95%CI: 19.1% - 19.3%); p < 0.001].

Thus, in the ICU, for every 100 patients with hemodynamic instability who suffer CA with non-shockable rhythms, 6 present ROSC. Of these, 4 die during hospitalization due to hemodynamic impairment after CA or due to severe neurological damage, and 2 are discharged. Among the patients discharged, 1.6 is able to recover their ability to perform ADLs. In turn, when CA occurs with shockable rhythms and in a patient without hemodynamic instability, the scenario is much more encouraging, with approximately 17 patients recovering their ability to perform ADLs for every 100 patients with CA treated.

## ADVANCED DISCUSSIONS ON DO-NOT-RESUSCITATE

Associated with statistical and prognostic issues, ethical questions arise when we face situations of withdrawal of life support or no escalation of care. The act of not resuscitating falls within the domain of no escalation of care.

In 1992, the implementation of international programs was instituted to assess the level of care in ICUs, aiming to identify the attitudes of health professionals with regard to cardiorespiratory resuscitation plans and administration, maintenance or removal of advanced life support in critically ill patients.^(18)^ The programs were based on the ethical principle of respect for patient autonomy, suggesting that life-support interventions were more appropriate when consistent with patient values rather than targeting specific organ dysfunction. The physician must be committed not only to the decision-making process but also to the patient's or their family's choice. Ideally, autonomy is the expression of the will of the patient, and in a simplistic view, to respect autonomy is to obey the will of the individual.^(19)^ However, adhering to the patient's choice should not result in disinterest, indifference and lack of empathy by medical personnel.^(20)^ Autonomy is the ability to freely decide, and free decision only exists when one is provided with all options. Consequently, communication is the fundamental pillar of autonomy, and it is essential that in the process of communication, all the information necessary for decision-making be provided by the physician without exerting undue pressure. Diseases limit freedom, and it is not appropriate, at the most vulnerable moments, for the individual to be allowed to give in to their desires. Moreover, having ensured successful communication, one should resist the temptation of blind obedience because respect does not mean total submission and does not require assent; it is not necessary for the doctor to agree with the patient's decision. In situations in which the patient cannot decide, the individuals responsible for the decision must, based on the patient's life history, try to suppose what the patient would do in that scenario. In these cases, the capacity of moral imagination is what will enable one to overcome simple obedience.^(21)^ The recognition of the tradition and the legacy of each individual can open the way for a decision that is appropriate for his/her history and life.

The importance of patient autonomy increases at a time when confidence in a positive outcome is lost, at least in the physician and in health institutions.^(22)^ The feeling of trust derives, in this case, from the competence, reliability and honesty of those who are dealing with the patient. The path taken to acquire trust, to show one is trustworthy, is misguided, and the greatest sign of credibility is vulnerability. Bureaucratization, through documents such as informed consent forms, and the unconditional respect for autonomy can be seen as mechanisms of protection of the professionals and the system, which may result in an increase in doubt as to what is being proposed by the physician. Before trust, we seek reliability, and basically, we have to prove ourselves trustworthy. How is this possible? Again, this is achieved through appropriate communication, which involves honesty, availability, clarity, empathy and commitment. To do good is to establish adequate communication because the starting point of ethics and justice is dialog. Most unwise decisions come from failure in dialog, and the primary failure is to assume that the other understood what was said.^(23)^

Suffering is determined by facts and not by communication. ICU admission is, by itself, a catalyst for suffering because it forces the individual to face several different dimensions. In addition to physical pain, there is fear of the immediate future and a search for meaning.^(24)^ The individual is faced with loneliness, and guilt and regret emerge. Finally, there is the fear of death or, more so, the despair over dying. Paradoxically, during life, we avoid this thought, the finality, which, now, in the ICU, becomes clear as never before. Reflections arising from suffering call into question the identity, prior choices and responsibility of others.^(25)^ There is a fear of the new human being who might emerge. With different intensities, these issues plague the patient and their family members.^(26,27)^ The clinical situation of the patient, especially the state of consciousness, is a determinant of his/her participation in this process. Cook et al.^(18)^ showed that half of those evaluated in the first 24 hours after admission to the ICU had an explicit desire not to be resuscitated. However, patients dependent on invasive ventilatory support in approximately 90% of the cases were unable to participate in the decision-making process.

Under no circumstances should autonomy be used as a political solution and nor aim to find harmony in an individualistic, plural and religious society. Autonomy should not become the cult of a private morality, and autonomous choices should be made considering duties, obligations and responsibilities - premises that apply to both the patient and the physician.^(28)^ However, the social and institutional contexts can be complicated because they can distort thoughts, desires and motivations. Thus, the stronger the connections between individuals, the more individual behavior will be similar to that of society, causing the patient to have a sense of powerlessness to go against the system, and the result may be the prevalence of the autonomy of the environment where the patient is inserted - a fact that is observed daily in hospitals and allows concluding that management options are not a means but an end in itself, and at the end, the autonomy of the technique ends up prevailing.^(29)^ The hospital imposes itself on the individual, trapping his/her soul and, thus, restricts the freedom of the patient in making decisions.

Asking the patient or his/her family if it is appropriate to use CPR maneuvers is an invitation for all those involved to participate in the decision-making process. Dialog presents the possibility of winning trust and establishing a relationship, which allow justice, correct actions and ethical decisions. Moral reflection propitiates ethical action, which, in turn, should be selfless; however, bioethics (ethics applied to health) addresses practical questions, and its deliberative character may not promote a broad reflection on the morality of actions, when one runs the risk of morality being adapted to the necessary action. Advanced discussions, particularly those about do-not-resuscitate orders, should be designed to promote self-determination because severely ill patients lose decision-making capacity. In addition, the wishes of family members and physicians do not necessarily match the wishes of patients. Thus, for patients requiring ICU admission, decisions on resuscitation in the event of CA are particularly important and should be explicit, so as to formalize a resuscitate or do-not-resuscitate plan.^(18)^ When no explicit directive is provided, i.e., there is no discussion on the subject and no information in medical records, the standard guideline is to perform CPR regardless of whether this intervention is consistent with patient values. Notably, however, in Brazilian ICUs, decision-making regarding do-not-resuscitate occurs, at times, without formal discussion on the subject and without an explicit directive recorded in the medical records, and consequently, the patient may or may not be resuscitated. In this context, the importance of implementing discussions on the use of CPR maneuvers is apparent.

International ICU care assessment programs suggest that advanced discussions about CPR should be conducted in the first 24 hours after ICU admission.^(30)^ Cook et al.,^(18)^ in an evaluation of 15 ICUs in 4 countries (n = 2,916), showed that only 11% of the patients had explicit discussions with the ICU team about resuscitation (50% with a resuscitate order and 50% with a do-not-resuscitate order). Limited functional status (OR, 4.8) and being unemployed (OR, 5.5) prior to ICU stay were associated with having a discussion about resuscitation. In these patients, a do-not-resuscitate order was influenced by patient age (OR, 8.8 for ≥ 75 years old), functional dependence prior to admission to the ICU (OR, 6.2), admission to the ICU when physicians are on duty or on the weekends and the inability of the patient to participate in the discussion (OR, 3.7). The authors suggest that this decision should be the responsibility of the ICU staff, rather than the physicians on duty. In addition, the authors demonstrated that physicians tend to overestimate the risk of death in the ICU, which ultimately influences the provision and limitation of life-support treatments.

The physician, when initiating the do-not-resuscitate discussion, places a new and objective element in the overall picture. The physician should start this conversation, but when and how. When should this conversation start? This should be initiated as soon as possible, i.e., before determining the general prognosis, particularly with patients at greater risk because, faced with high morbimortality, it is important for options to be offered and for the situation to be treated with seriousness. Time has to be an ally of those involved in the decision-making process. How should this conversation start? This is more delicate, but basically, the physician has to create the right setting, ensuring enough time and privacy; preferentially involve the patient in the dialog, talking with him/her and the family together, when possible; assess if the timing is appropriate; be confident regarding the information that they want to transmit and prepare in advance a simple and understandable explanation about the prognosis of the underlying disease, e.g., what is CA, and the resuscitation maneuvers; inform, clearly and calmly; be emotionally supportive; and, at the end, confirm that all participants understood the information and plan the next steps.^(31,32)^ It is not uncommon for further communication and new explanations to be necessary because denial and memory issues are the most common responses to existential suffering.^(33)^ We are entering a territory far beyond medical knowledge, and if we think of medicine exclusively as a technical profession, we are doomed to failure because our primary objective is to relieve suffering in all its manifestations. We think of medical practice as an art, that is, a set of actions that are marked by a particular personality, capacity and intelligence. Consequently, medicine cannot be reduced only to techniques. Suffering is part of living, and the physician must be humble and recognize the limitations, and monumental task, regarding relieving all pain because suffering can become little more than a logical puzzle when approached from a safe distance.^(34)^ The empathic attitude and the adequate response to the needs of the patient and their loved ones require health professionals to distance themselves - a distance that does not make them indifferent to the suffering but far enough so that the professional avoids emotional identification.^(35)^ Importantly, the patient is never safe from torment, and his/her relatives share the existential suffering with the patient, which encompasses the recognition of one's own mortality, deprivation of freedom, loneliness and the perception of a meaningless life. The physician is the vehicle for information that can increase suffering, but he/she also has the capacity to relieve suffering. It is important not to confuse the messenger, the physician, with the message, the bad news, because it is not the transmitted information that causes pain - pain rises from the situation that affects patients and their families. Communication, when done properly, is therapeutic.^(36)^

## FINAL CONSIDERATIONS

Advanced discussions on do-not-resuscitate in cases of CA in the ICU indicate good medical practices, good organization of the unit, clarity of definitions and, most important of all, respect for the patient. The discussion includes guiding the patient and family during decision-making. We have to use persuasion, avoiding coercion and manipulation. Persuading someone implies promoting a change in conduct through the strength of sound arguments, without disrespecting the values of those being helped and without giving up one's responsibility as a helper. Persuasion can be considered the effort needed to impart scientific knowledge based on the reality of the patient; it is the art of making the truth apparent. Coercion and manipulation imply the restriction of freedom of choice and preclude autonomous decisions.^(37)^

Prognostic estimates, even when based on robust data, should be provided with caution because population data are difficult to apply in specific cases. Although quantitative estimates of survival help patients make decisions regarding CPR, they strongly value quality of life after successful CPR.^(38)^ For example, 80% of patients opted for CPR when the chance of survival was estimated as no more than 10%.^(38)^ In addition, quality of life after survival was considered "extremely important" by more than one-third of patients.^(39)^

Information should always be recorded as thoroughly as possible and should be recorded based on the 5 E's of medical practice, analogous with the constructivist model of education. First, *e*ngage and then *e*xamine, including complementary examinations. During the process, *e*vidence will be evaluated, and *e*xplanations will be empathically and clearly provided. Last, the physician will write it down. Omission of decisions regarding CPR in the medical record should be the exception, not the rule. The lack of definition represents the inability of the physician to communicate effectively with the patient/family, aiming at an alignment of expectations regarding the patient's prognosis. It attests at most superficial knowledge of the subject and reflects the difficulty of a deeper approach to the problem, as the prognosis of CRP in critically ill patients is very poor. In short, communication represents a bridge between medical personnel and patients for approaching such a complex, inconvenient issue, avoids the appearance of disregard by the professional for the patient and their relatives, and demonstrates the ability of the professional to assume his/her responsibilities towards the care of the patient.

So, advanced discussions regarding do-not-resuscitate are based on the ethical principle of respect for patient autonomy because the wishes of family members and physicians do not necessarily match those of patients. In addition to the promotion of autonomy, advanced discussions can help the medical and care team anticipate future problems, allowing better patient care. Our opinion is that discussions on resuscitation of critically ill patients should be performed for all patients within the first 24 to 48 hours after admission to the intensive care unit.
